# Supplementary material for: Acetylcholine waves and dopamine release in the striatum
Source: Nat Commun. 2023 Oct 27;14:6852. doi: 10.1038/s41467-023-42311-5 (PMC10611775; doi:10.1038/s41467-023-42311-5)
Supplement: Supplementary file 1 — Supplementary Information [file 41467_2023_42311_MOESM1_ESM.pdf]

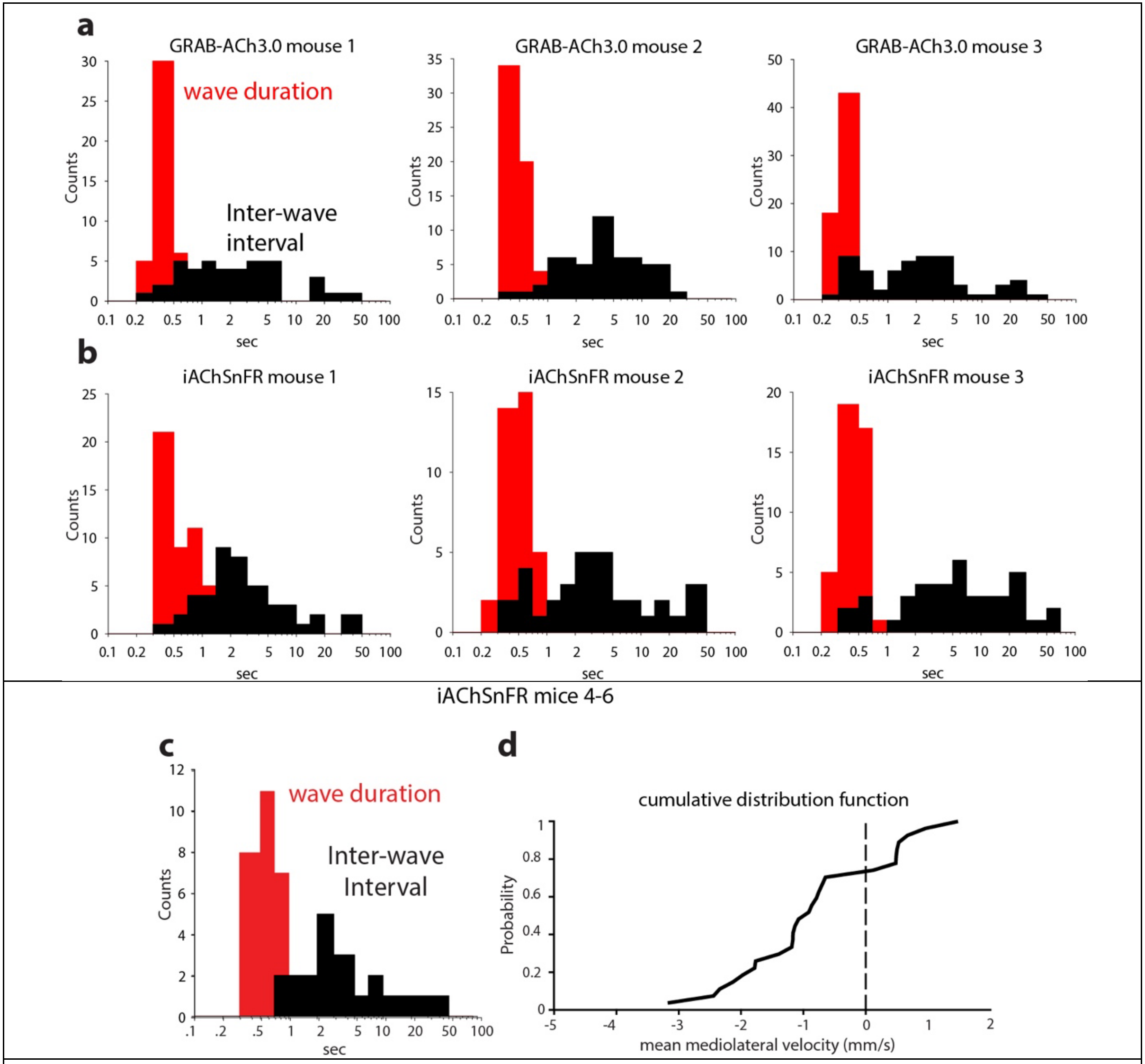

Supplementary Fig. 1: **a**. Distributions of wave durations and inter-wave intervals pooled from individual mice imaged with GRAB-ACh3.0. **b**. Distributions of wave durations and inter-wave intervals pooled from individual mice (1-3) imaged with iAChSnFR. **c**. Distributions of wave durations and inter-wave intervals pooled from mice 4-6 imaged with iAChSnFR. d. Distributions of wave velocities pooled from mice 4-6 imaged with iAChSnFR. Source data are provided as a Source Data file.

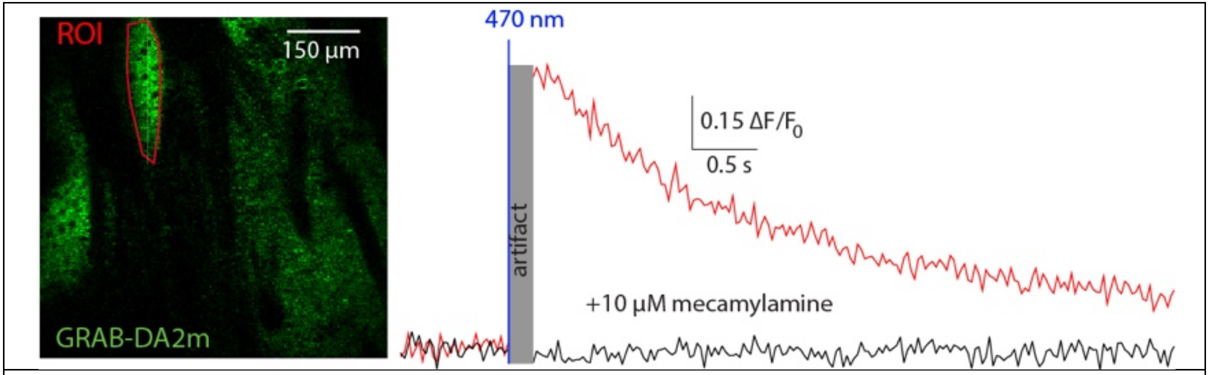

Supplementary Fig. 2a: Synchronous optogenetic activation (with a 1-ms long, 470 nm pulse) of CINs in an acute slice from a ChAT-ChR2 mouse whose dorsal striatum was inoculated with AAVs harboring GRAB-DA2m, causes DA release that is eliminated in the presence of 10 µM mecamylamine, a nAChR antagonist. DA release was evoked in 8 slices from 4 mice, and the mecamylamine block was tested and confirmed in 3 slices from two of these mice. The artifact which is removed is explained in the Methods section.

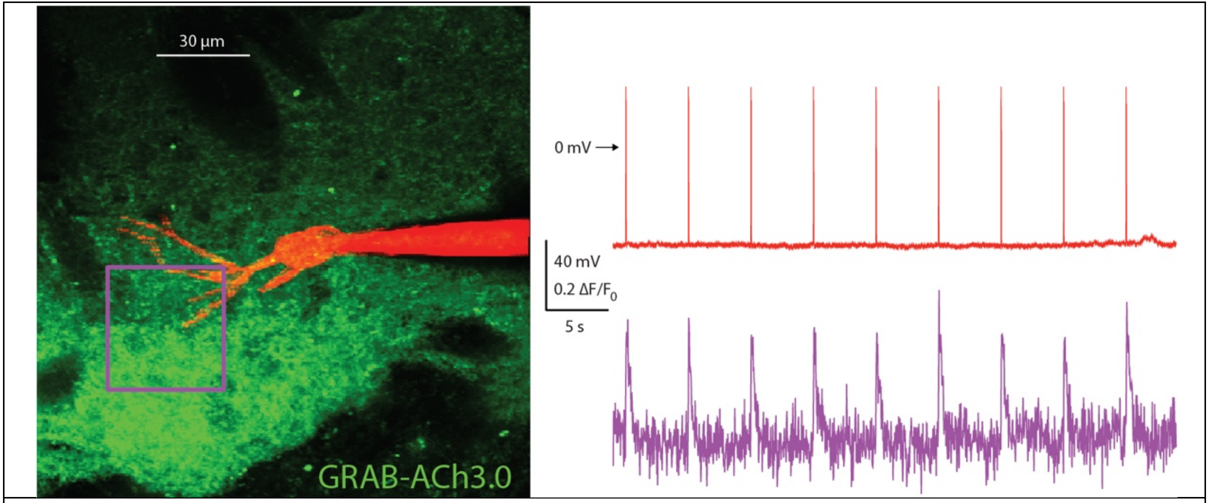

Supplementary Fig. 2b: Nine action potentials were elicited in an individual CIN hyperpolarized to quiescence (each with a 35 ms-long, 220 pA current pulse). The action potentials triggered ACh release (averaged from the color coded box) as visualized in wild-type mice whose dorsal striatum was inoculated with GRAB-ACh3.0. At this frequency (0.2 Hz) the median decrease of the amplitude of the 2nd to 9th ACh responses was no more than 4% relative to the 1st response (*n = 4* CINs from *N = 3* mice).

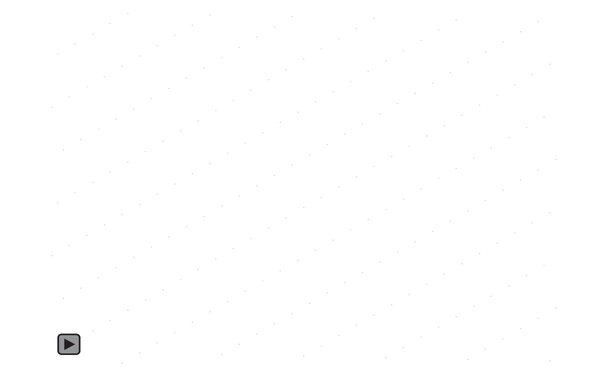	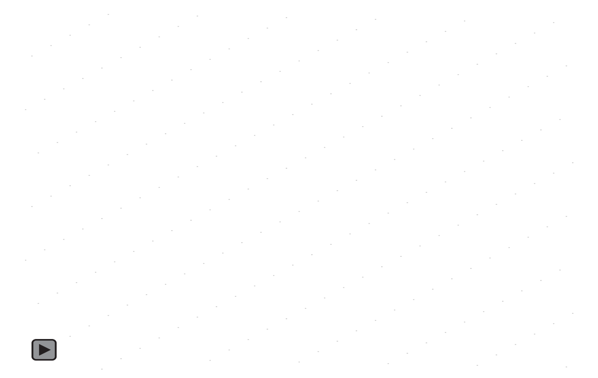
$\beta=1$, $D_u=0.02$ (T=50)	$\beta=1.8$, $D_u=0.02$ (T=50)
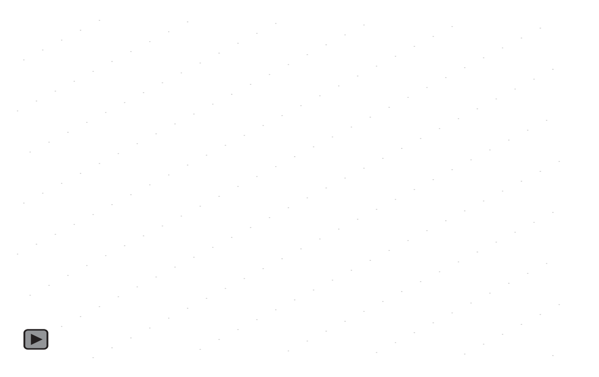	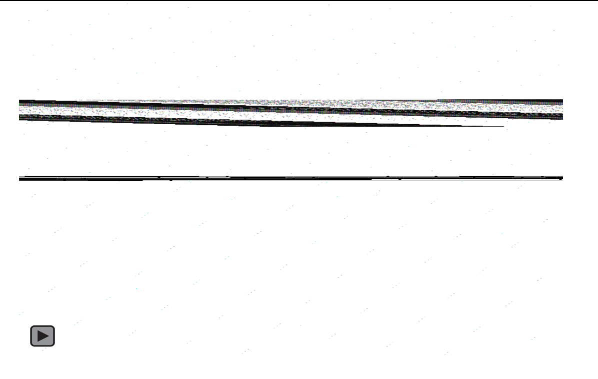
$\beta=1$, $D_u=1$ (T=10)	$\beta=1.8$, $D_u=1$ (T=10)
**Supplementary Fig. 3a**: Full model with $f_1(u)$ and $g_1(u)$. red: $u(x,t)$ (CIN); blue: $v(x,t)$ (DA). Other parameters: $A=4.2$, $\sigma=0.75$, $\kappa=1.5$, $\gamma=0.47$, $\phi = 10$, $D_v=1$. Length of simulation in time units is indicated in parentheses.	

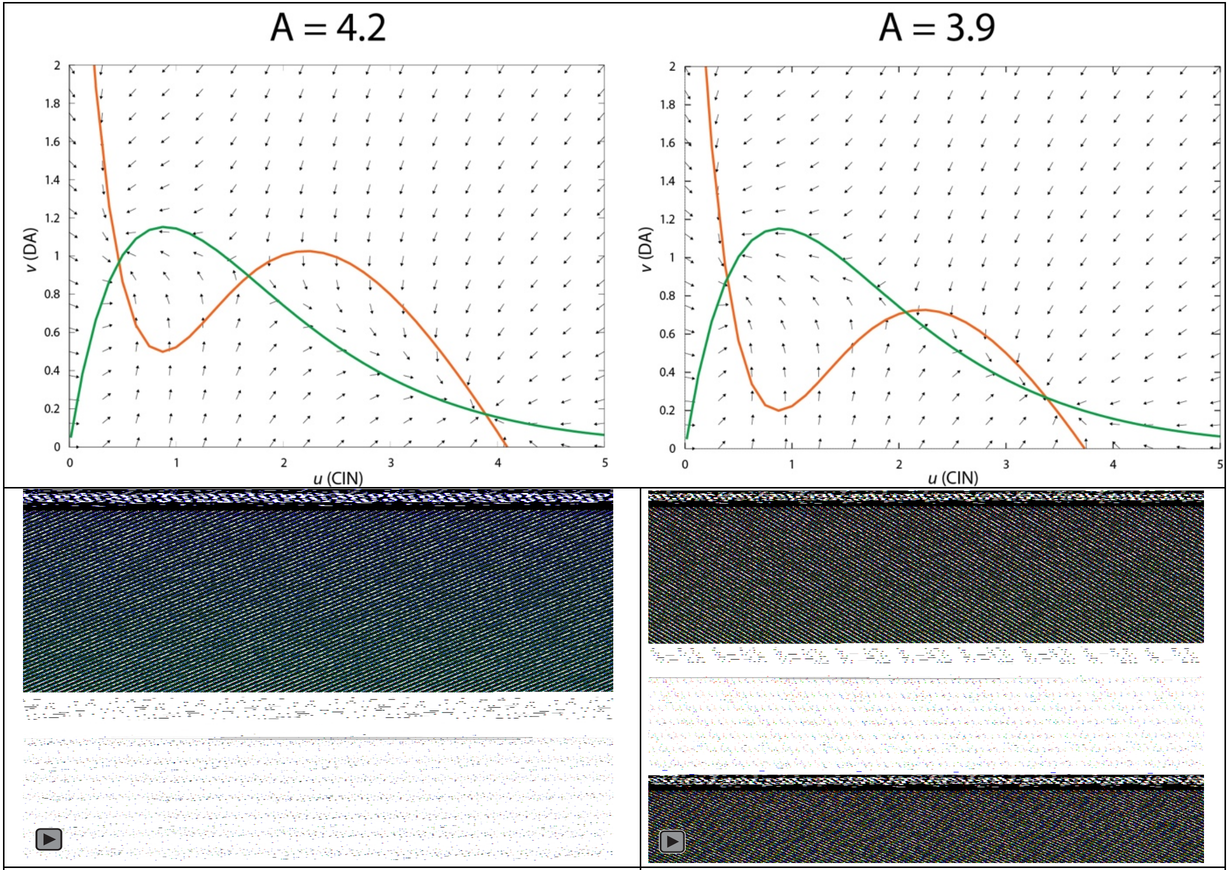

A=4.2 (T=50)	*A=3.9* (T=50)

Supplementary Fig. 3b: Full model with $f_1(u)$ and $g_1(u)$. red: $u(x,t)$ (CIN); blue: $v(x,t)$ (DA). Other parameters: $\beta=1.2$, $\sigma=0.75$, $\kappa=1.5$, $\gamma=0.47$, $\phi = 10$, $D_u=0.02$, $D_v=1$. Length of simulation in time units is indicated in parentheses.

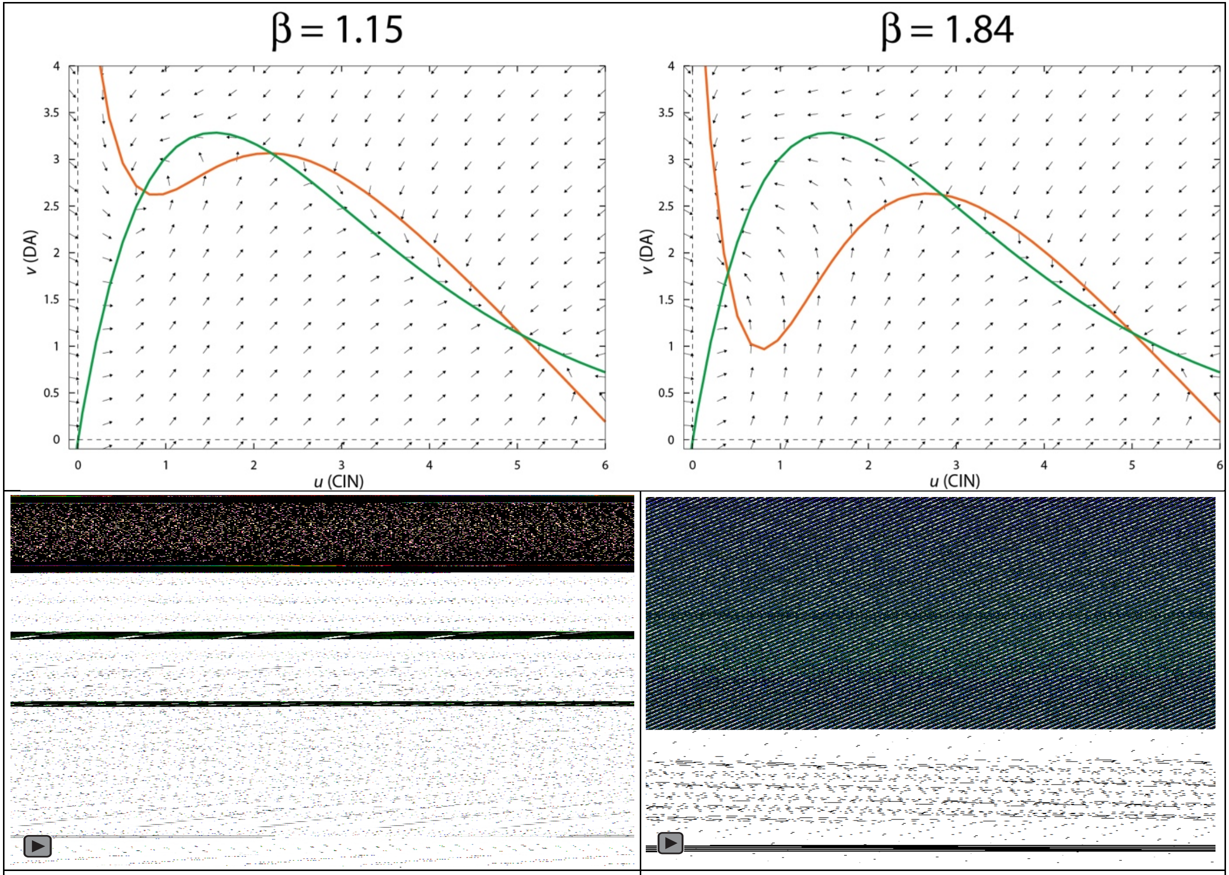

β=1.15, D_v=0 (T=300)	*β=1.84, D_v=0* (T=100)

Supplementary Fig. 3c: Full model with $f_1(u)$ and $g_1(u)$ with $D_v=0$. red: $u(x,t)$ (CIN); blue: $v(x,t)$ (DA). Other parameters: A=*6.2*, $\sigma=0.43$, $\kappa=1.5$, $\gamma=1.34$, $\phi = 10$, $D_u=0.1$. Length of simulation in time units is indicated in parentheses.

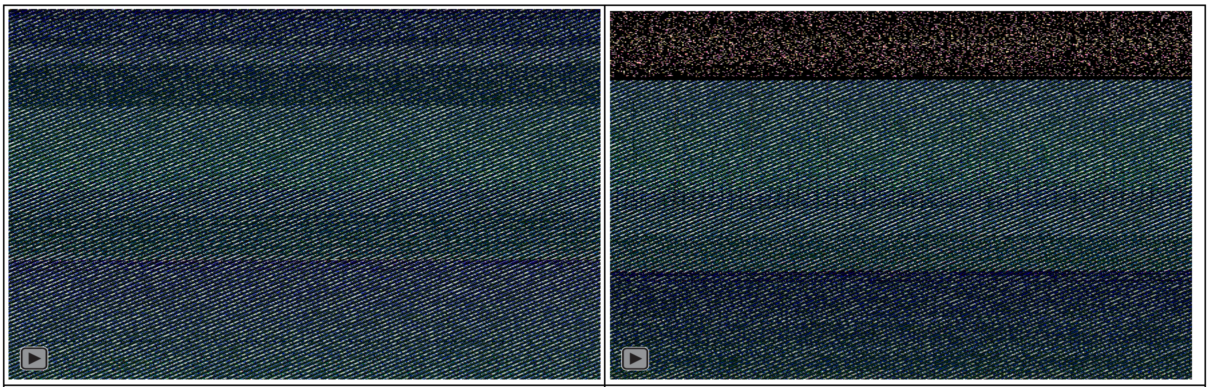

b=0.435	*b=0.7*

Supplementary Fig. 4: Tractable model with $f_2(u)$ and $g_2(u)$ and $D_v=0$. red: $u(x,t)$ (CIN); blue: $v(x,t)$ (DA). Other parameters: $a=0.3$, $s=0.2$, $D_u=0.01$.

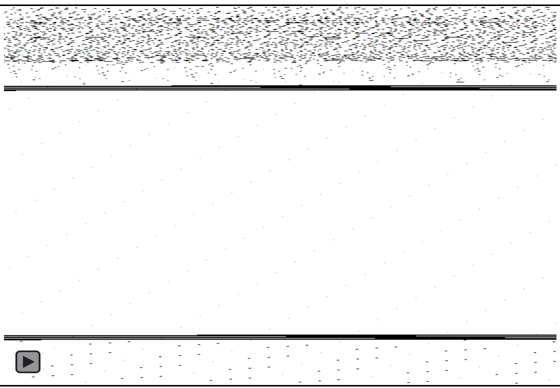

Supplementary Fig. 5: Turing instability in tractable model with $f_2(u)$ and $g_2(u)$. red: $u(x,t)$ (CIN); blue: $v(x,t)$ (DA). Other parameters: $a=0.3$, $b=0.435$, $s=0.2$, $D_u=0.06$, $D_v=1$.

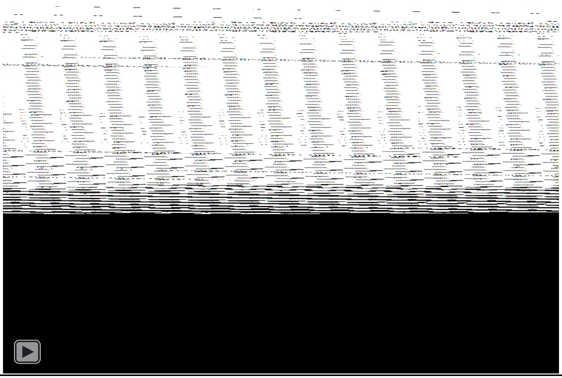

$v(x_o, t=20) = 15$

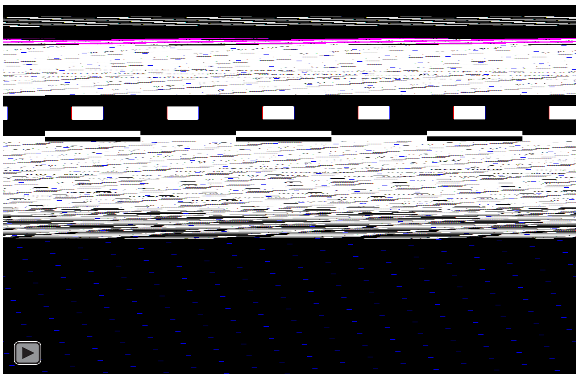

$v(x_o, t=20) = 20$

Supplementary Fig 6a. Response to transient local activation of v (DA) in full model with $f_1(u)$ and $g_1(u)$. red: $u(x,t)$ (CIN); blue: $v(x,t)$ (DA). Other parameters: $A=3.9$, $\beta=1.2$, $\sigma=0.75$, $\kappa=1.5$, $\gamma=0.47$, $\phi = 10$, $D_u=0.01$, $D_v=1$.

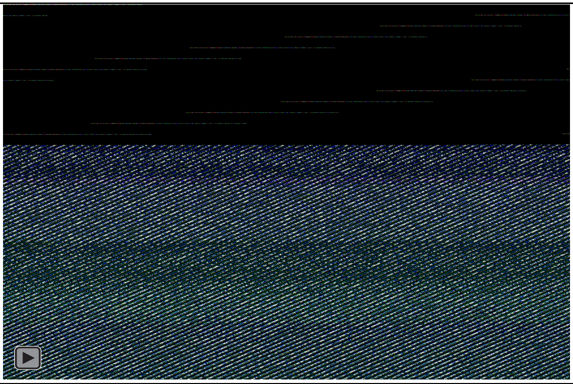

$u(x_o, t=5) = 10$

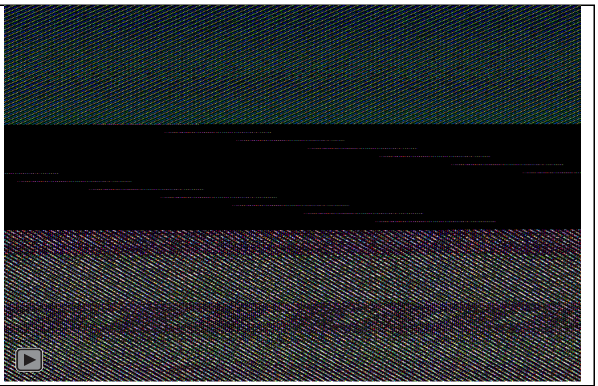

$u(x_o, t=5) = 20$

Supplementary Fig 6b. Response to transient local activation of u (CIN) in full model with $f_1(u)$ and $g_1(u)$. red: $u(x,t)$ (CIN); blue: $v(x,t)$ (DA). Other parameters: $A=4.3$, $\beta=1.2$, $\sigma=0.75$, $\kappa=1.5$, $\gamma=0.47$, $\phi = 10$, $D_u=0.01$, $D_v=1$.

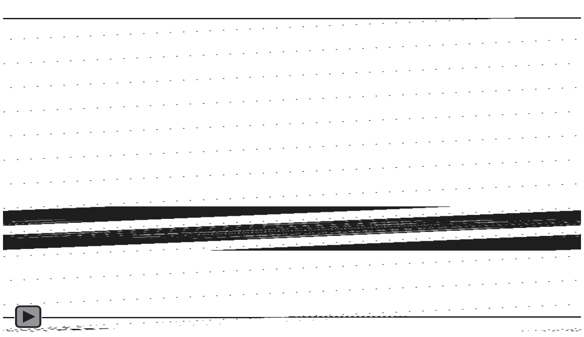	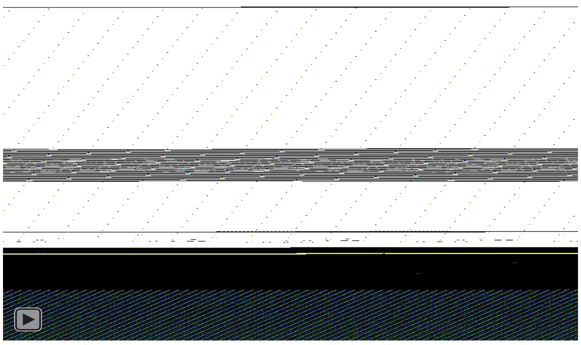
β=1.35	*β=1.55*

Supplementary Fig 7. Full model with $f_1(u)$ and $g_1(u)$ with $\sigma=0.1$. red: $u(x,t)$ (CIN); blue: $v(x,t)$ (DA). Other parameters: $A=4.3$, $\kappa=1.5$, $\gamma=0.47$, $\phi = 10$, $D_u=0.2$, $D_v=1$.

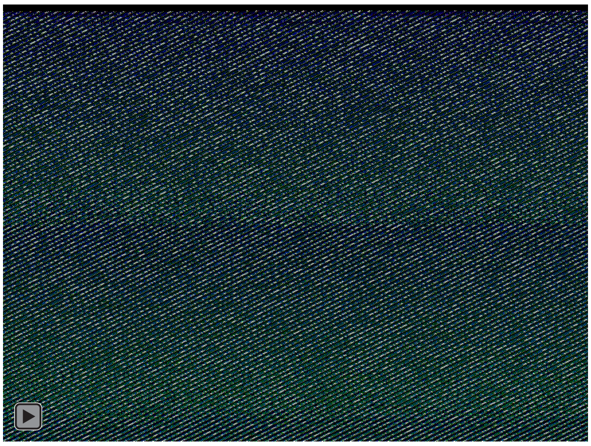
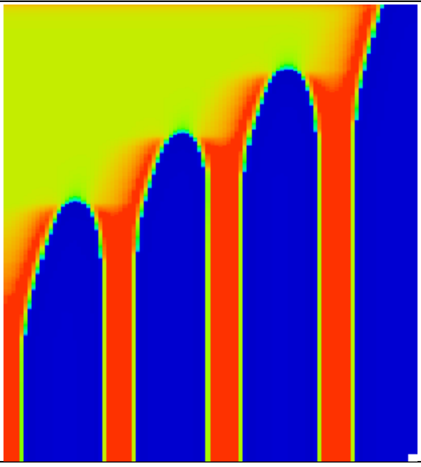

D_u=0.01

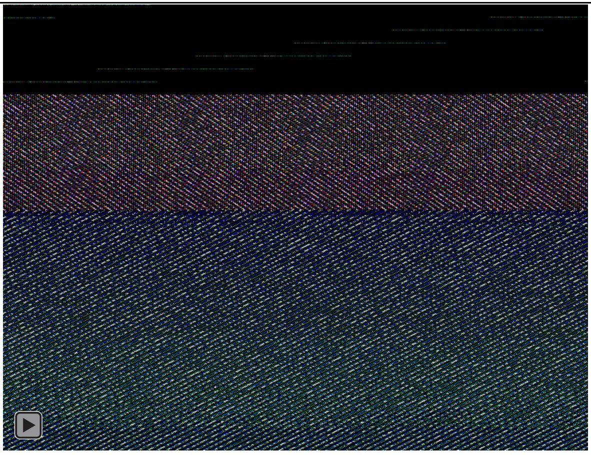
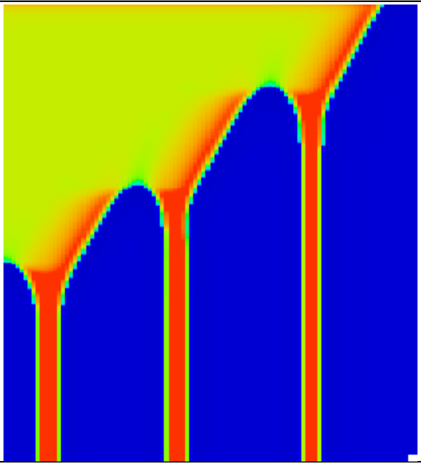

D_u=0.015

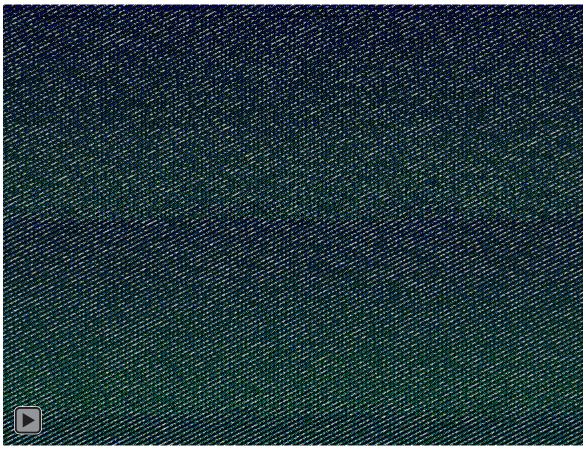
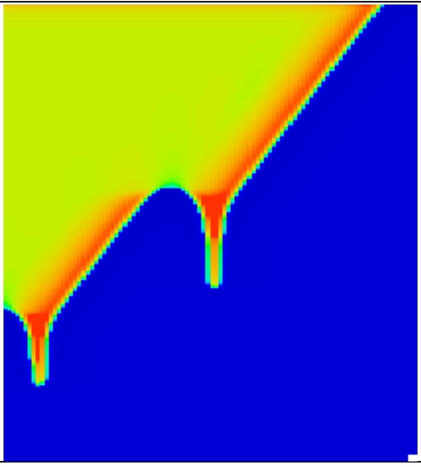

D_u=0.02

Supplementary Fig. 8: Stable and unstable Turing patterns in full model with $f_1(u)$ and $g_1(u)$. red: $u(x,t)$ (CIN); blue: $v(x,t)$ (DA). Other parameters: A=4.3, β=1.62, σ=0.1, κ=1.5, γ=0.5, D_v=1.

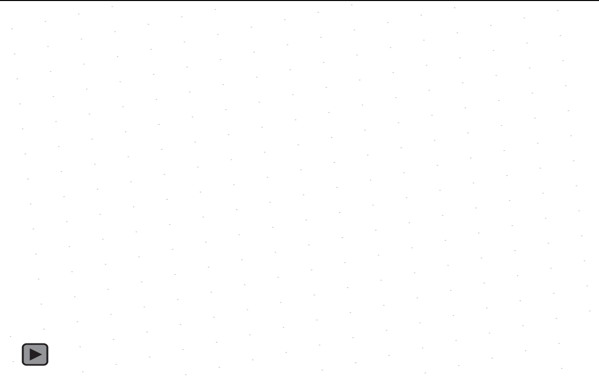	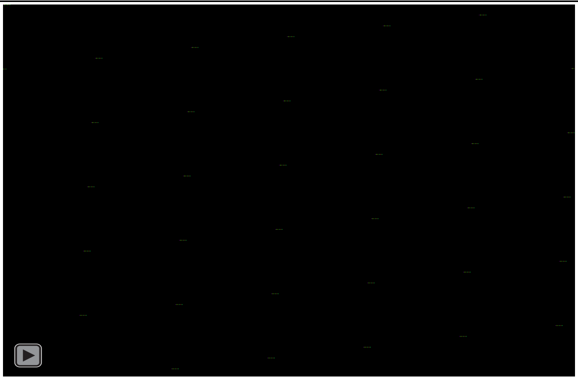
s=0.1	*s=0.25*

Supplementary Fig 9. Tractable model with $f_3(u)$ and $g_3(u)$. red: $u(x,t)$ (CIN); blue: $v(x,t)$ (DA). Other parameters: $b=0.14$, $D_u=0.1$, $D_v=1$.

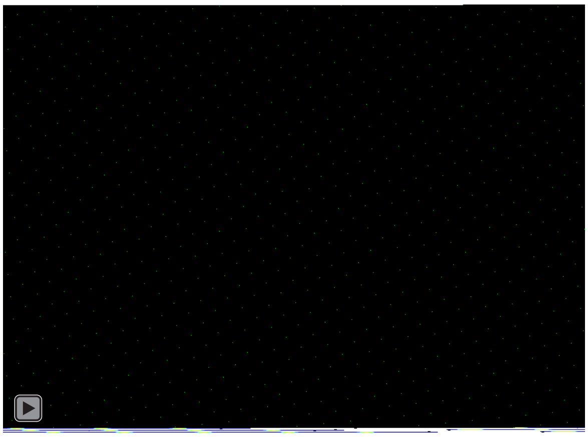 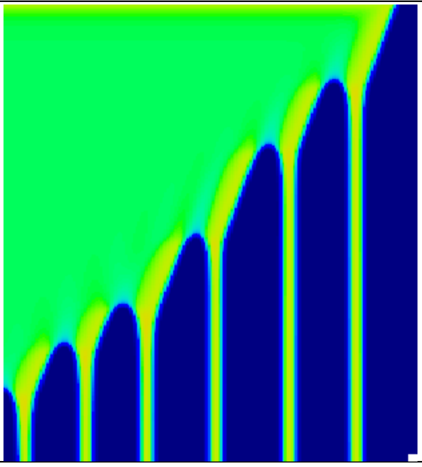

D_u=0.02

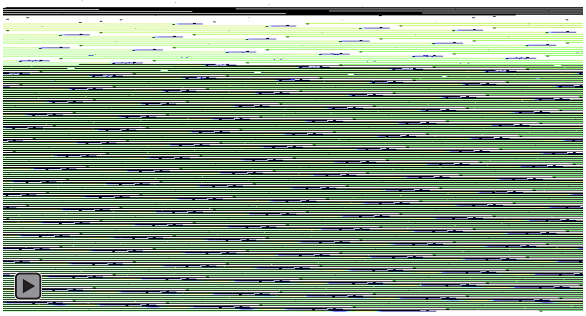 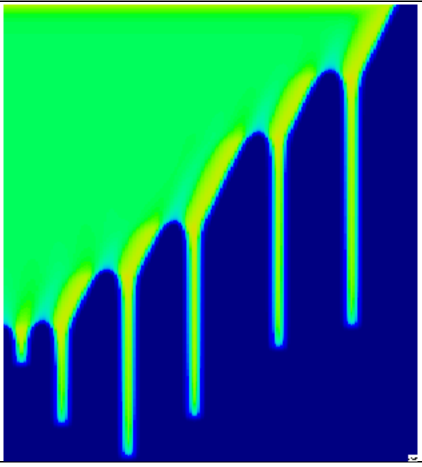

D_u=0.022

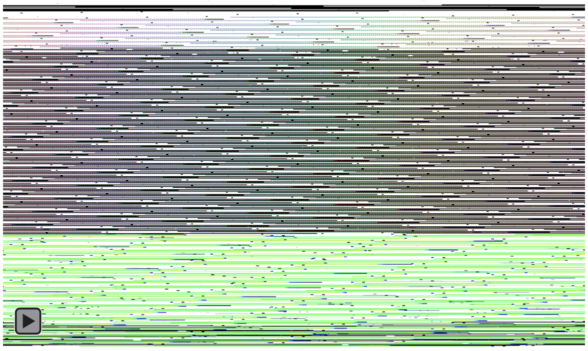 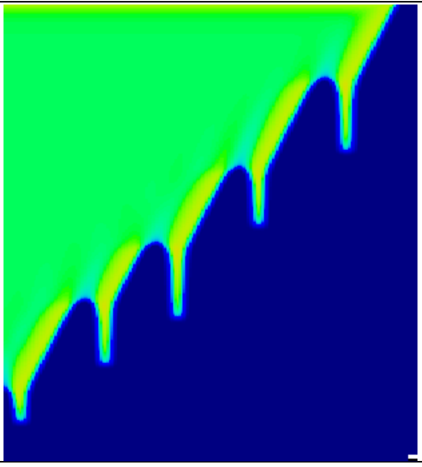

D_u=0.024

Supplementary Fig. 10: Stable and unstable Turing patterns in tractable model with $f_3(u)$ and $g_3(u)$. red: $u(x,t)$ (CIN); blue: $v(x,t)$ (DA). Other parameters: $s=0.25$ $b=0.14$, $D_v=1$.
