# Supplementary material for: Acetylcholine waves and dopamine release in the striatum
Source: Nat Commun. 2023 Oct 27;14:6852. doi: 10.1038/s41467-023-42311-5 (PMC10611775; doi:10.1038/s41467-023-42311-5)
Supplement: Supplementary file 3 — Description of Additional Supplementary Files [file 41467_2023_42311_MOESM3_ESM.pdf]

Description of Additional Supplementary Files

File Name: Supplementary Movie 1
Description: Top: Acetylcholine waves in the fluorescence ($\Delta F/F0$) of GRAB-ACh3.0 that is expressed in the dorsal striatum of a mouse as viewed through a 3 mm diameter cranial window. Bottom: Space–time rendition of the wave activity collapsed along the mediolateral aspect of the image (See Fig. 1 in the Results). The central line corresponds to the frame shown above. Black points mark the location of the maximal activity along the mediolateral aspect. Movie slowed down by a factor of 12.5.

File Name: Supplementary Movie 2
Description: Top: Acetylcholine waves in the fluorescence ($\Delta F/F0$) of iAChSnFR that is expressed in the dorsal striatum of a mouse as viewed through a 1 mm diameter GRIN lens. Bottom: Space–time rendition of the wave activity collapsed along the mediolateral aspect of the image (See Fig. 1 in the Results). The central line corresponds to the frame shown above. Black points mark the location of the maximal activity along the mediolateral aspect. Movie slowed down by a factor of 5.

File Name: Supplementary Code
Description: Code for running the simulations used in the manuscript. Please consult the README.txt file included in the zipped file.
