# Supplementary material for: Acetylcholine waves and dopamine release in the striatum
Source: Nat Commun. 2023 Oct 27;14:6852. doi: 10.1038/s41467-023-42311-5 (PMC10611775; doi:10.1038/s41467-023-42311-5)
Supplement: Supplementary file 6 — Supplementary Code [file 41467_2023_42311_MOESM6_ESM.zip › Code/AChDA.ani.rtf]

# animation for the array# TuringAChDA.ani# next for original file f1 g1#fcircle .005+.01*[0..100];.25*u[j];.01;$RED#fcircle .005+.01*[0..100];.25*v[j];.01;$BLUEfcircle .005+.01*[0..100];.18*u[j];.01;$REDfcircle .005+.01*[0..100];.18*v[j];.01;$BLUE#next for f2 g2#fcircle .005+.01*[0..100];.95*u[j];.01;$RED#fcircle .005+.01*[0..100];.95*v[j];.01;$BLUE# next for f3 g3#fcircle .005+.01*[0..200]/2;u[j];.01;$RED#fcircle .005+.01*[0..200]/2;1*v[j];.01;$BLUEend
