# Supplementary material for: Acetylcholine waves and dopamine release in the striatum
Source: Nat Commun. 2023 Oct 27;14:6852. doi: 10.1038/s41467-023-42311-5 (PMC10611775; doi:10.1038/s41467-023-42311-5)
Supplement: Supplementary file 7 — Reporting Summary [file 41467_2023_42311_MOESM7_ESM.pdf]

nature portfolio

Corresponding author(s): Joshua A. Goldberg

Last updated by author(s): Sep 19, 2023

Reporting Summary

Nature Portfolio wishes to improve the reproducibility of the work that we publish. This form provides structure for consistency and transparency in reporting. For further information on Nature Portfolio policies, see our Editorial Policies and the Editorial Policy Checklist.

Statistics

For all statistical analyses, confirm that the following items are present in the figure legend, table legend, main text, or Methods section.

n/a	Confirmed	
☐	☒	The exact sample size (*n*) for each experimental group/condition, given as a discrete number and unit of measurement
☐	☒	A statement on whether measurements were taken from distinct samples or whether the same sample was measured repeatedly
☐	☒	The statistical test(s) used AND whether they are one- or two-sided<br>*Only common tests should be described solely by name; describe more complex techniques in the Methods section.*
☒	☐	A description of all covariates tested
☒	☐	A description of any assumptions or corrections, such as tests of normality and adjustment for multiple comparisons
☐	☒	A full description of the statistical parameters including central tendency (e.g. means) or other basic estimates (e.g. regression coefficient) AND variation (e.g. standard deviation) or associated estimates of uncertainty (e.g. confidence intervals)
☐	☒	For null hypothesis testing, the test statistic (e.g. *F*, *t*, *r*) with confidence intervals, effect sizes, degrees of freedom and *P* value noted<br>*Give P values as exact values whenever suitable.*
☒	☐	For Bayesian analysis, information on the choice of priors and Markov chain Monte Carlo settings
☐	☒	For hierarchical and complex designs, identification of the appropriate level for tests and full reporting of outcomes
☒	☐	Estimates of effect sizes (e.g. Cohen's *d*, Pearson's *r*), indicating how they were calculated

Our web collection on statistics for biologists contains articles on many of the points above.

Software and code

Policy information about availability of computer code

Data collection	Two photon imaging: Femto2D Galvo and resonant scanner, running MES and MESc.
Data analysis	Matlab was used for rudimentary arithmetic calculations (means, medians, etc); statistical hypothesis testing; and for simulating data for bootstrapping, which is explained in detail in the manuscript.

For manuscripts utilizing custom algorithms or software that are central to the research but not yet described in published literature, software must be made available to editors and reviewers. We strongly encourage code deposition in a community repository (e.g. GitHub). See the Nature Portfolio guidelines for submitting code & software for further information.

Data

Policy information about availability of data

All manuscripts must include a data availability statement. This statement should provide the following information, where applicable:

- Accession codes, unique identifiers, or web links for publicly available datasets
- A description of any restrictions on data availability
- For clinical datasets or third party data, please ensure that the statement adheres to our policy

Source data are provided with the paper. The raw data generated in this study belonging to Figs. 1-3 and Suppl. Figs. 1-2 have been deposited in Dryad https://doi.org/10.5061/dryad.b5mkkwhk8.

Human research participants

Policy information about studies involving human research participants and Sex and Gender in Research.

Reporting on sex and gender	N/A
Population characteristics	N/A
Recruitment	N/A
Ethics oversight	N/A

Note that full information on the approval of the study protocol must also be provided in the manuscript.

Field-specific reporting

Please select the one below that is the best fit for your research. If you are not sure, read the appropriate sections before making your selection.

☒ Life sciences ☐ Behavioural & social sciences ☐ Ecological, evolutionary & environmental sciences

For a reference copy of the document with all sections, see nature.com/documents/nr-reporting-summary-flat.pdf

Life sciences study design

All studies must disclose on these points even when the disclosure is negative.

Sample size	In vivo imaging (Figure 1): 3 mice were use for GRAB-ACh imaging at NYU. 6 mice were used for iAChSnFR imaging at OIST.<br>Acute slice imaging (Figure 2 and Suppl. Fig. 2a): GRAB-DA2m data in response to electrical stimulation were gathered from a total of 11 brain slices from 2 mice. GCaMP6f data were from a total of 15 slices from 3 mice. GRAB-DA2m data in response to full-field optogenetics were gathered form a total of 8 brain slices from 4 mice.<br>Acute slice electrophysiology and imaging (Figure 3 & Suppl Fig. 2b): GRAB-DA2m data were gathered from a total of 41 CINs from 18 mice. GRAB-ACh3.0 data were gathered from a total of 4 CINs from 3 mice.<br>The above sample sizes were used in order to replicate the data in at least 3 mice, as is standard in our field. The only exception is the experiments to estimate the spatial falloff of the dopamine release (Figure 2c), where due to the nested design of the study, we found that our effect was significant with 2 mice (P = 0.025) using the linear mixed-effect model (LMEM), and we therefore did not need to use an additional mouse.
Data exclusions	The only data excluded were the movies from one mouse injected (in the OIST cohort) with iAChSnFR, where the signal was too poor for analysis (and was from opposite hemisphere to all other 9 mice used). This was not a pre-established exclusion.
Replication	We used 2-6 mice in each cohort, with multiple imaging sessions or brain slices per mouse. All attempts at replication were successful. The only exception was the experiments where we describe a new phenomenon of eliciting DA release by activating a single CIN. These were repeated in 18 mice. The new phenomenon was observed in 10 of them. Both the number of repetitions and the number of cases showing the new phenomenon were reported.
Randomization	N/A, because according to the study design we were not comparing between treatments/groups. We were only gathering new physiological data describing novel phenomena within the same group (Figs. 1&3), or comparing within subjects (Fig. 2c).
Blinding	There was no group allocation in the study design, because we were not comparing groups. In the experiment, where detecting an event was the dependent variable (Fig. 3), a colleague, who did not collect the data, determined the presence or absence of an event.

Reporting for specific materials, systems and methods

We require information from authors about some types of materials, experimental systems and methods used in many studies. Here, indicate whether each material, system or method listed is relevant to your study. If you are not sure if a list item applies to your research, read the appropriate section before selecting a response.

Materials & experimental systems

n/a	Involved in the study
☒	☐ Antibodies
☒	☐ Eukaryotic cell lines
☒	☐ Palaeontology and archaeology
☐	☒ Animals and other organisms
☒	☐ Clinical data
☒	☐ Dual use research of concern

Methods

n/a	Involved in the study
☒	☐ ChIP-seq
☒	☐ Flow cytometry
☒	☐ MRI-based neuroimaging

Animals and other research organisms

Policy information about studies involving animals; ARRIVE guidelines recommended for reporting animal research, and Sex and Gender in Research

Laboratory animals	Most experiments were carried out in C57BL/6J mice (Strain #:000664; Jackson Laboratories, Bar Harbor, ME, USA), with three exceptions. In the iAChSnFR experiments we used ChAT-IRES-Cre (Δneo) transgenic mice (stock number 031661; Jackson Laboratories, Bar Harbor, ME, USA). In the experiments monitoring the activity of CINs in brain slices, we used ChAT-IRES-Cre (Δneo) transgenic mice cross-bred to mice expressing Cre-dependent, Tet-controllable GCaMP6f (Ai148, TIT2L-GC6f-ICL-tTA2; stock number 030328; Jackson Laboratories, Bar Harbor, ME, USA). For experiments in which we studied the release of DA in response to synchronous activation of CINs, we cross-bred the ChAT-IRES-Cre (Δneo) mice with mice expressing Cre-dependent channelrhodopsin-2 [Ai32, RCL-ChR2(H134R)/EYFP; stock number 012569; Jackson Laboratories, Bar Harbor, ME, USA]. Two-to-seven-months-old C57BL/6J and transgenic mice were used for experiments. All mice were housed under a 12-h light/dark cycle with food and water ad libitum. Ambient temperatures and humidity were 22 ± 2C and 50 ± 10%, respectively.
Wild animals	No wild animals were used in the study.
Reporting on sex	Based on similar experiments in other laboratories, sex was not considered as a factor in the design of the experiments, and so mice of both sexes were used, and their results pooled. The sex of the mice used is listed in the Source Data file.
Field-collected samples	No field collected samples were used in the study.
Ethics oversight	Experimental procedures adhered to and received prior written approval from the Institutional Animal Care and Use Committees of the Hebrew University of Jerusalem (HUJI, protocol number MD-20-16113-3), the Okinawa Institute of Science and Technology (OIST, protocol number 2021-336-2) and the New York University Grossman School of Medicine (NYUGSOM, protocol number IA16-02082).

Note that full information on the approval of the study protocol must also be provided in the manuscript.
